# Accuracy Bounds for Array-Based Positioning in Dense Multipath Channels †

**DOI:** 10.3390/s18124249

**Published:** 2018-12-03

**Authors:** Thomas Wilding, Stefan Grebien, Ulrich Mühlmann, Klaus Witrisal

**Affiliations:** 1 Signal Processing and Speech Communication Laboratory, Graz University of Technology, 8010 Graz, Austria; 2 Christian Doppler Laboratory for Location-aware Electronic Systems, Graz University of Technology, 8010 Graz, Austria; stefan.grebien@tugraz.at (S.G.); witrisal@tugraz.at (K.W.); 3 NXP Semiconductors, 8101 Gratkorn, Austria; ulrich.muehlmann@nxp.com

**Keywords:** indoor positioning, wireless positioning, CRLB, AoA, ToA, antenna-array signal processing

## Abstract

The accuracy of radio-based positioning systems will be limited by multipath interference in realistic application scenarios. This paper derives closed-form expressions for the Cramér–Rao lower bound (CRLB) on the achievable time-of-arrival (ToA) and angle-of-arrival (AoA) estimation-error variances, considering the presence of multipath radio channels, and extends these results to position estimation. The derivations are based on channel models comprising deterministic, specular multipath components as well as stochastic, diffuse/dense multipath. The derived CRLBs thus allow an evaluation of the influence of channel parameters, the geometric configuration of the environment, and system parameters such as signal bandwidth and array geometry. Our results quantify how the ToA and AoA accuracies decrease when the signal bandwidth is reduced, because more multipath will then interfere with the useful LoS component. Antenna arrays can (partly) compensate this performance loss, exploiting diversity among the multipath interference. For example, the AoA accuracy with a 16-element linear array at 1MHz bandwidth is similar to a two-element array at 1GHz, in the magnitude order of one degree. The ToA accuracy, on the other hand, still scales by a factor of 100 from the cm-regime to the m-regime because of the dominating influence of the signal bandwidth. The position error bound shows the relationship between the range and angle information under realistic indoor channel conditions and their different scaling behaviors as a function of the anchor–agent placement. Specular multipath components have a maximum detrimental influence near the walls. It is shown for an L-shaped room that a fairly even distribution of the position error bound can be achieved throughout the environment, using two anchors equipped with 2×2-array antennas. The accuracy limit due to multipath increases from the 1–10-cm-range at 1GHz bandwidth to the 0.5–1-m-range at 100MHz.

## 1. Introduction

High-accuracy positioning will be a key enabler for a wide range of novel applications in different sectors of industry, including manufacturing, logistics, retail, and transportation. Outdoors, global navigation satellite systems have become the backbone of such location-aware systems [1], exploiting the time-of-flight of radio signals, while indoors, a wide range of wireless systems has been considered [2]. The focus on radio technologies is due its advantages, e.g., small size, low cost, and low power consumption, however its performance is ultimately limited by physical properties of the radio environment, in particular by multipath propagation. Signal reflections at objects lead to fading and distortion, which both influence the performance of ranging and positioning systems [3].

Positioning with radio signals can be achieved with a number of measurement methods [4], exploiting different parameters of the received radio signals, e.g., the received signal strength (RSS), time-(difference-)of-arrival (ToA/TDoA) and angle-of-arrival (AoA). RSS and AoA can be used with narrow bandwidth (BW) signals, however, their performance is influenced heavily by multipath fading [5]. A higher BW, in particular ultra-wide-bandwidth (UWB) signals, can be utilized to diminish the effects of multipath fading and distortion [3]. Finally, the position estimation requires multiple anchors for multiangulation, multilateration, or joint use of time and angle information enabling single-anchor positioning. For ToA estimation, a tight synchronization is needed between the anchors and the agents or a specific ranging protocol, e.g., two-way ranging [4]. For AoA estimation, a synchronized array is needed at the receiver (achieving phase coherence) but the demand on synchronization between anchors and agents is less stringent [6].

One possibility to analyze the performance of a positioning system and the influence of its parameters (e.g., signal bandwidth and geometric configuration), is to derive statistical performance bounds. A popular bound is the Cramér–Rao lower bound (CRLB), a lower bound on the achievable variance of any unbiased estimator [7]. CRLB analyses can be classified as: (i) measurement-based CRLBs; or (ii) waveform-based CRLBs. Measurement-based CRLBs are derived from statistical models of position-related measurements extracted from received signals, e.g., the ToA, TDoA, AoA, or RSS. Therefore, measurement-based CRLBs depend heavily on the specific models used for the position-related parameters and may thus neglect relevant information for localization [8]. Waveform-based CRLBs start at the received signal waveforms and enable to highlight the influence of signal and system parameters directly on the derived bounds. The CRLB derivations presented in this work belong to the class of waveform-based bounds, allowing to statistically characterize the impact of interfering multipath components.

An overview of measurement-based CRLBs can be found in [9,10,11] which includes comparisons with a large number of estimators. In [12,13], a CRLB analysis of ToA based positioning in multipath is given for line-of-sight (LoS) and non-line-of-sight (NLoS) scenarios, showing that NLoS propagation can improve the positioning accuracy when prior knowledge is available. Measurement-based bounds for ToA, TDoA, AoA and RSS positioning were compared in [14], showing that AoA and RSS schemes suffer most from increasing the anchor–agent distance.

Regarding the waveform-based class, a generic overview of different bounds is given in [15] and specifically for time-delay-estimation in [16]. In the following, we list references belonging to this class, beginning with single-antenna anchors and agents, and moving towards array-based studies.

Waveform-based CRLBs for positioning in additive white Gaussian noise (AWGN) channels are evaluated in [17] for round-trip ToA, ToA and TDoA systems, showing that ToA outperforms the other two and highlighting the sensor geometry dependence. In [18], the effect of path-overlap between multipath components is quantified by the ranging ability outage, accounting for the influence of different pulse shapes. A thorough framework for positioning in terms of the CRLB, evaluating the contribution of prior information of the channel parameters, NLoS propagation and the effect of clock asynchronism was described in [8].

In [19,20], the influence of multipath interference is investigated by introducing an additional term in the channel model, called dense multipath (DM), which allows for an evaluation of the impact of multipath interference. The influence of the signal bandwidth is investigated in [19], while the potential utilization of reflected, specular multipath components (MPCs) to increase the achievable positioning accuracy, is considered in [20,21]. The stochastic IEEE 802.15.4a UWB channel model is evaluated in [3] in terms of the expectable ranging performance.

In the field of array-based positioning, the waveform-based CRLB for joint ToA and AoA radar positioning in combination with optimal sensor placement was examined in [22], as well as for UWB signals in terms CRLB expressions for hybrid ToA-AoA positioning in [23]. The CRLB for positioning including agent movement and showing the contribution of the Doppler shift to the obtainable AoA information was examined in [24]. In [8,25], the influence of unknown array orientation on the resulting positioning accuracy is examined, allowing the inclusion of prior information about position, orientation and multipath parameters. In [26], a polarimetric signal model is used to derive general expressions for the waveform-based CRLB for channel parameter estimation including dense multipath components, but the results were not analyzed with respect to the position estimation problem. Most recently, the performance of massive-MIMO systems equipped with mm-wave antenna arrays at the agent and anchor sides has been studied [27,28,29], evaluating aspects such as the orientation estimation accuracy, the difference between uplink and downlink channels, the potential use of MPCs, and different transceiver frontend configurations.

In this paper, we extend our previous work on performance limits for high-accuracy localization in multipath channels [19,20] towards antenna arrays and joint ToA and AoA estimation by deriving the waveform-based CRLB. We further examine the resulting achievable positioning accuracy to be expected in dense multipath channels, exploiting these ToA and AoA estimates.

We derive a canonical expression for the Fisher information matrix (FIM), which allows a deeper insight into the dependence on the signal and system parameters, e.g., bandwidth, SNR, or number of array elements.

**Figure 1:**
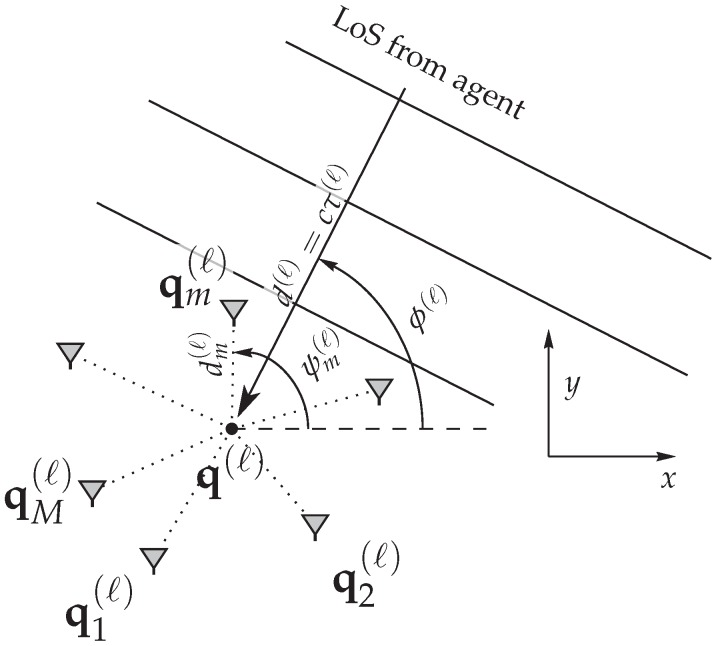
Geometry of the array at the anchor position $q^{\left(\ell \right)}$ (reference point) with positions of the *m*th array element $q_{m}^{\left(\ell \right)}$, described by the angle $\psi_{m}^{\left(\ell \right)}$ and the distance $d_{m}^{\left(\ell \right)}$ of element *m* from the reference point $q^{\left(\ell \right)}$. $\phi^{\left(\ell \right)}$ is the AoA and $\tau^{\left(\ell \right)}$ the ToA from an agent at a position $p={\left[\;x,y\;\right]}^{T}$.

A geometric description of the generic antenna array as employed throughout this work is shown in Figure 1. We stress that arbitrary array geometries can be used for the derivations, as long as the assumptions introduced later on remain valid. Furthermore, the analyzed signal model is very generic and can be adapted to many signaling schemes, as long as the transmitted pulse shape is known. Thus, the results are not limited to UWB signals, but are also applicable to other modulating schemes, including direct-sequence-spread-spectrum and OFDM.

A possible application of these accuracy bounds is to analyze and quantify the positioning performance limits for specific system setups, using a wide range of wireless technologies, e.g., the IEEE 802.15.4 UWB standard, the upcoming 5G systems exploiting mm-wave or massive MIMO, global navigation satellite systems, and Internet-of-things devices.

Thus, we address the following research questions:

- How does dense multipath influence the position estimation based on ToA and AoA estimation?
- How do the system and signal parameters influence the ToA, AoA and position estimation in presence of dense multipath?
- How does “path-overlap” by specular components influence the position estimation (based on line-of-sight ToAs and AoAs) in presence of dense multipath?

The main novel aspects we present are:

- We formulate the CRLB for array-based AoA estimation in presence of dense multipath (DM) and compare the CRLB for ToA estimation in such channels, providing insight on the impact of system parameters (e.g., bandwidth, antenna configuration, and carrier frequency) and radio channel parameters. (This novel aspect was previously presented at the ICL-GNSS in 2018 [30].)
- We analyze and evaluate the position error bound for a multi-anchor scenario based on joint ToA and AoA estimation, showing the trade-off between these two measurement parameters and their scaling behaviors with respect to bandwidth and anchor–agent geometry.
- We formulate and analyze “path-overlap”, the interference of the useful LoS component by specular MPCs, and illustrate its impact with respect to the environment geometry.

The paper is structured as follows: In Section 2, we introduce the signal model, including the array processing for the LoS and the DM process. In Section 3, we derive the CRLB for ranging and angulation. Next, we numerically evaluate these two bounds, the ranging error bound (REB) and the angulation error bound (AEB) and discuss the influence of the DM process and system parameters on these bounds. In Section 4 we derive the CRLB for positioning called position error bound (PEB). Furthermore, we extend the signal model to include multiple specular components and DM and derive the PEB again to include the effect of path-overlap. Then, we analyze and discuss the PEB for different positioning schemes, including AoA-only, ToA-only and a combined AoA-ToA version. Finally, Section 5 concludes the paper by summing up the findings.

## 2. Signal Model

We consider the case of a single agent at an unknown position $p={\left[\;x,y\;\right]}^{T}$ in Cartesian coordinates which transmits a signal to an anchor *ℓ* at known position $q^{\left(\ell \right)}={\left[\;x_{a}^{\left(\ell \right)},y_{a}^{\left(\ell \right)}\;\right]}^{T}$. (The theoretical framework described in this paper can be straightforwardly modified to allow for different Tx–Rx configurations.) Similar to the signal models in [19,24], the received signals are modeled as complex baseband equivalent signals, allowing coherent processing of the signals at each antenna element due to the explicit inclusion of the carrier phase. A known wideband signal s(t)∈R transmitted from an agent to the anchor *ℓ* yields the received signal at array element m∈1,⋯,M, with *M* denoting the total number of antennas [20]

(1) $$\begin{array}{cc} r_{m}^{\left(\ell \right)}\left(t\right) & =a_{m}^{\left(\ell \right)}s\left(t-\tau_{m}^{\left(\ell \right)}\right)+\left(s*\nu_{m}^{\left(\ell \right)}\right)\left(t\right)+w_{m}\left(t\right) \end{array}$$

where the first term models the deterministic line-of-sight (LoS) component with complex-valued amplitude $a_{m}^{\left(\ell \right)}\in C$ and time delay $\tau_{m}^{\left(\ell \right)}$ at the antenna elements, and *c* is the speed of light. The array geometry is depicted in Figure 1 and described in detail in Section 2.1.

The amplitude $a_{m}^{\left(\ell \right)}$ is decomposed as

(2) $$\begin{array}{cc} a_{m}^{\left(\ell \right)} & ={\bar{\alpha }}_{m}^{\left(\ell \right)}e^{-j\left(2\pi f_{c}\tau_{m}^{\left(\ell \right)}+\varphi^{\left(\ell \right)}\right)}=\alpha_{m}^{\left(\ell \right)}e^{-j2\pi f_{c}\tau_{m}^{\left(\ell \right)}} \end{array}$$

where ${\bar{\alpha }}_{m}^{\left(\ell \right)}\in R^{+}$ is the absolute value of the amplitude and the phase is separated into delay-dependent and random phase parts. The latter represents the unknown initial phase of the agent and any influences on the phase due to the antenna responses or other effects. We use $j=\sqrt{-1}$ as the imaginary unit. We assume that the array aperture is small enough that the amplitude differences at each antenna element *m* are negligibly small (Assumption S1), i.e., ${\bar{\alpha }}_{m}\approx \bar{\alpha }$ described by the approximation

(3) $$\begin{array}{cc} a_{m} & \approx {\bar{\alpha }}^{\left(\ell \right)}e^{-j\left(2\pi f_{c}\tau_{m}^{\left(\ell \right)}+\varphi^{\left(\ell \right)}\right)}=\alpha^{\left(\ell \right)}e^{-j2\pi f_{c}\tau_{m}^{\left(\ell \right)}}, \end{array}$$

with $\alpha^{\left(\ell \right)}\in C$.

The second term in Equation (1) accounts for all occurring reflections, representing the dense multipath [19,20]. It is modeled by the convolution of the transmit signal s(t) with a random process $\nu_{m}^{\left(\ell \right)}\left(t\right)$. To characterize the process, we use the assumption of uncorrelated scattering (Assumption S2) in the delay domain [31], through which the auto-correlation function of $\nu_{m}^{\left(\ell \right)}\left(t\right)$ can be written as

(4) $$\begin{array}{cc} E_{\nu }\;\left\{\nu_{m}^{\left(\ell \right)}\left(t\right){\left[\nu_{m^{\prime }}^{\left(\ell \right)}\left(u\right)\right]}^{*}\right\} & =S_{\nu }^{\left(\ell \right)}\left(t-\tau_{m}^{\left(\ell \right)}\right)\delta \;\left(t-u\right)\delta \left[m-m^{\prime }\right]. \end{array}$$

In this equation, $\delta \;\left(\cdot \right)$ is the Dirac delta, δ[·] is the Kronecker delta, and $S_{\nu ,m}\left(t\right)$ describes the power delay profile (PDP) of the DM at array element *m*. For simplicity, it is assumed that the DM is uncorrelated between antenna elements *m* and $m^{\prime }$ (Assumption S3), which is a valid assumption under conditions of λ/2-spaced linear arrays and uniformly distributed scatterers in 3D [32]. It is furthermore assumed that the DM is quasi-stationary in the spatial domain, i.e., the PDP does not change in the vicinity of $q^{\left(\ell \right)}$ the DM only depends on the agent position p and anchor reference point $q^{\left(\ell \right)}$, resulting in an identical PDP shape at each array element (Assumption S4). Similar to Witrisal et al. [19], we model the DM process $\nu_{m}^{\left(\ell \right)}\left(t\right)$ by a zero-mean Gaussian process.

The last term in Equation (1), $w_{m}\left(t\right)$, describes additive white Gaussian noise with double-sided power spectral density of $N_{0}/2$.

In this section, we introduced Assumptions (S1)–(S4). When we invoke one of these assumptions in the following sections, we reference to the specific assumptions.

### 2.1. Relation to Array Geometry

An overview of the array geometry is shown in Figure 1, which is similar to [24]. For simplicity—and in accordance with many practical scenarios—we assume that anchor and agent are located on a plane, removing the need for estimating the elevation angle. The extension to three dimensions in terms of environment as well as arrays is straightforward. For some given array, the time delay, corresponding to the received signals $r_{m}^{\left(\ell \right)}\left(t\right)$ at element *m*, is related to the array geometry by

(5) $$\begin{array}{c} \tau_{m}^{\left(\ell \right)}=\tau^{\left(\ell \right)}-\frac{d_{m}^{\left(\ell \right)}\cos\left(\phi^{\left(\ell \right)}-\psi_{m}^{\left(\ell \right)}\right)}{c} \end{array}$$

where $\tau^{\left(\ell \right)}=d^{\left(\ell \right)}/c$ is the time delay, termed time-of-arrival (ToA), from the agent location p to an arbitrarily chosen array reference point $q^{\left(\ell \right)}$ related to the range $d^{\left(\ell \right)}={∥q^{\left(\ell \right)}-p∥}_{}$. The AoA $\phi^{\left(\ell \right)}$ is measured with respect to the known array orientation and given by $\phi^{\left(\ell \right)}=∠\left(q^{\left(\ell \right)}-p\right)$. The distance and angle of element *m* from the reference point $q^{\left(\ell \right)}$ are $d_{m}^{\left(\ell \right)}$ and $\psi_{m}^{\left(\ell \right)}$, respectively. The position of an array element $q_{m}^{\left(\ell \right)}$ from the reference point is

(6) $$\begin{array}{c} q_{m}^{\left(\ell \right)}=q^{\left(\ell \right)}+d_{m}^{\left(\ell \right)}\cdot \left[\begin{array}{c} \cos\;(\psi_{m}^{\left(\ell \right)})\\ \sin\;(\psi_{m}^{\left(\ell \right)}) \end{array}\right]. \end{array}$$

As shown below, the most practical choice of the reference point is the center of gravity of the antenna element positions (see also [23,25]).

For the sake of a more concise notation, we omit the anchor index *ℓ* in the following, proceeding with a single-anchor scenario, and reintroduce it when needed.

## 3. Cramér–Rao Lower Bound for AoA and ToA

A popular measure for evaluating estimator performance is the Cramér–Rao lower bound (CRLB) which gives a lower bound on the achievable variance of any unbiased estimator. Before we examine the position error bound (PEB), we perform a separate evaluation of the ranging error bound (REB) and the angulation error bound (AEB).

The general form of the Fisher information matrix (FIM) for a parameter vector θ that parameterizes the probability density $p\;\left(r;\theta \right)$ for an observation r becomes [7,33]

(7) $$\begin{array}{c} J_{\theta }=E_{r;\theta }\;\left\{\left(\frac{\partial }{\partial \theta }\ln\;p\;\left(r;\theta \right)\right){\left(\frac{\partial }{\partial \theta }\ln\;p\;\left(r;\theta \right)\right)}^{T}\right\} \end{array}$$

from which the CRLB for an estimator $\hat{\theta }$ of said parameter vector is defined as

(8) $$\begin{array}{c} E_{\theta }\;\left\{\left(\hat{\theta }-\theta \right){\left(\hat{\theta }-\theta \right)}^{T}\right\}⪰J_{\theta }^{-1}. \end{array}$$

The operation A⪰B on two matrices indicates that the matrix A−B is positive semidefinite.

(9) $$\begin{array}{cc} p\;\left(r;\theta \right) & =\prod_{m=1}^{M}p\;\left(r_{m};\theta \right). \end{array}$$

The parameter vector for the setup as shown in Figure 1 is $\theta ={\left[\;\phi ,\tau ,R\alpha ,I\alpha \;\right]}^{T}\in R^{4}$, where Rα and Iα are the real and imaginary parts of the LoS amplitude α∈C.

Under the Gaussian model, the likelihood function for the received signal vector $r_{m}$ at array element *m* is

(10) $$\begin{array}{cc} p\;\left(r_{m};\theta \right) & \propto \exp\;\{-{\left(r_{m}-a_{m}s_{m}\right)}^{H}C_{m}^{-1}\left(r_{m}-a_{m}s_{m}\right)\} \end{array}$$

where $C_{m}=\sigma_{n}^{2}I_{N}+C_{c,m}\in R^{N\times N}$ is the overall covariance matrix of the sampled noise processes at element *m*, $I_{N\times N}$ is an N×N identity matrix and $\sigma_{n}^{2}=\frac{N_{0}}{T_{s}}$ is the noise variance. The covariance of the DM component can be written in matrix notation as $C_{c,m}=SS_{\nu ,m}S^{H}$, where $S={\left[{\bar{s}}_{0},{\bar{s}}_{1},\cdots ,{\bar{s}}_{N-1}\right]}^{T}$ is the convolution matrix of the sampled pulses ${\bar{s}}_{i}={\left[\;s\left(iT_{s}\right),s\left(\left(i-1\right)T_{s}\right),\cdots ,s\left(\left(i-N+1\right)T_{s}\right)\;\right]}^{T}\in R^{N}$ and $S_{\nu ,m}$ is the correlation matrix of the DM at element *m*, which is a diagonal matrix under the uncorrelated scattering (US) Assumption (S2).

Similar to Witrisal et al. [19], we use an eigendecomposition of the covariance matrix $C_{m}=U_{m}\Lambda_{m}U_{m}^{H}$ to write it as $C_{m}=\sum_{i=1}^{N}u_{i,m}u_{i,m}^{H}\left(\lambda_{i,m}+\sigma_{n}^{2}\right)$ where $u_{i,m}$ and $\lambda_{i,m}\in R$ are the *i*th eigenvector and corresponding eigenvalue of $C_{c,m}$ which make up the *i*th column of $U_{m}$ and diagonal element of $\Lambda_{m}$. We further introduce a weighted inner product defined as

(11) $$\begin{array}{cc} {\langle x,y\rangle }_{H_{m}} & =\sigma_{n}^{2}y^{H}C_{m}^{-1}x \end{array}$$

(12) $$\begin{array}{c} =\sum_{i=1}^{N}\frac{y^{H}u_{i,m}u_{i,m}^{H}x}{\lambda_{i,m}/\sigma_{n}^{2}+1} \end{array}$$

and the induced norm as ${∥x∥}_{H_{m}}^{2}={\langle x,x\rangle }_{H_{m}}$ for a Hilbert space $H_{m}$ characterized by a specific covariance matrix $C_{m}$ (see Appendix in [19]). The weighting can be interpreted as a whitening operation.

A straightforward result from Equations (3) and (4) is that the transmitted signals have equal induced norms, i.e., ${∥s_{m}∥}_{H_{m}}^{2}={∥s∥}_{H}^{2}\forall m$, due to the identical shape of the DM PDP at each element *m*. This allows us to omit the index *m* in $H_{m}$ with s being a sampled version of s(t−τ).

### 3.1. Fisher Information Matrix

The main ingredient to compute the CRLB is the Fisher information matrix, alongside some restrictions imposed on the likelihood function to be able to arrive at a closed form solution [7]. Inserting Equation (9) into Equation (7), we obtain the 4×4 Fisher information matrix $J_{\theta }$ for the parameter vector θ as

(13) $$\begin{array}{cc} J_{\theta } & =\sum_{m=1}^{M}J_{\theta ,m} \end{array}$$

which allows a separate examination of the information added by each array element. By separating the full FIM into sub-blocks for desired parameters ϕ and τ and nuisance parameters Rα and Iα, we obtain

(14) $$\begin{array}{c} J_{\theta }=\sum_{m=1}^{M}J_{\theta ,m}=\left[\begin{array}{cc} A & B\\ B^{T} & D \end{array}\right]\in R^{4\times 4} \end{array}$$

where the block matrices are

(15) $$\begin{array}{cc} A & =\sum_{m=1}^{M}\left[\begin{array}{cc} J_{\phi \phi ,m} & J_{\phi \tau ,m}\\ J_{\tau \phi ,m} & J_{\tau \tau ,m} \end{array}\right]=\left[\begin{array}{cc} J_{\phi \phi } & 0\\ 0 & J_{\tau \tau } \end{array}\right] \end{array}$$

(16) $$\begin{array}{cc} B & =\sum_{m=1}^{M}\left[\begin{array}{cc} J_{\phi R\alpha ,m} & J_{\phi I\alpha ,m}\\ J_{\tau R\alpha ,m} & J_{\tau I\alpha ,m} \end{array}\right]=\left[\begin{array}{cc} 0 & 0\\ J_{\tau R\alpha } & J_{\tau I\alpha } \end{array}\right] \end{array}$$

(17) $$\begin{array}{cc} D & =\sum_{m=1}^{M}\left[\begin{array}{cc} J_{R\alpha R\alpha ,m} & J_{R\alpha I\alpha ,m}\\ J_{I\alpha R\alpha ,m} & J_{I\alpha I\alpha ,m} \end{array}\right]=\left[\begin{array}{cc} J_{R\alpha R\alpha } & 0\\ 0 & J_{I\alpha I\alpha } \end{array}\right]. \end{array}$$

The zero elements of the block matrices are due to the chosen array reference point and the resulting contribution of each array element’s FIM [23], which is shortly demonstrated in Appendix A.

To gain insight into the parameters of interest, we use the equivalent Fisher information matrix (EFIM), which is well established in the literature [8,19,24]. As we focus on AoA and ToA estimation, we obtain the corresponding EFIM ${\left[J_{\theta }\right]}_{2\times 2}^{-1}$ for the truncated parameter vector $\theta_{1,2}={\left[\;\phi ,\tau \;\right]}^{T}$ by applying the Schur complement on Equation (14)

(18) $$\begin{array}{c} {\left[J_{\theta }\right]}_{2\times 2}^{-1}={\left(A-BD^{-1}B^{T}\right)}^{-1}={\left[\begin{array}{cc} J_{\phi } & 0\\ 0 & J_{\tau } \end{array}\right]}^{-1}. \end{array}$$

The connection between EFIM and CRLB is then

(19) $$\begin{array}{c} E_{\theta }\;\left\{{\hat{\theta }}_{1,2}-\theta_{1,2}){\left({\hat{\theta }}_{1,2}-\theta_{1,2}\right)}^{T}\right\}⪰{\left[J_{\theta }\right]}_{2\times 2}^{-1}. \end{array}$$

The structure of the FIM in Equation (14) indicates that the estimation of the ToA τ and AoA ϕ of the LoS component in DM are decoupled. The structure of B further shows that the estimation of the LoS amplitude α only influences the estimation of τ. Further insights are examined in the following sections in terms of the achievable bounds on ToA and AoA, as well as the resulting positioning accuracy in terms of the PEB.

### 3.2. Ranging Error Bound (REB)

By evaluating Equation (18), we obtain the ranging error bound (REB) $R=\sqrt{J_{\tau }^{-1}}$, which is the square root of the lower bound $\mathrm{var}\left\{\hat{\tau }\right\}\geq J_{\tau }^{-1}$ of the variance for estimating the time delay of the LoS component.

The result for the REB is very similar to the one shown in [19], the only difference being the scaling by the number of antenna elements *M*. The EFIM for the range estimation problem is

(20) $$\begin{array}{cc} J_{\tau } & =8\pi^{2}\beta^{2}\gamma_{\tau }\mathrm{SINR}\sin^{2}\left(\xi \right)M \end{array}$$

(21) $$\begin{array}{c} =8\pi^{2}\beta^{2}{\tilde{\mathrm{SINR}}}_{\tau }M, \end{array}$$

where $\beta^{2}$ is the mean-squared bandwidth [34] defined as $\beta^{2}={∥\dot{s}∥}^{2}/\left(4\pi^{2}{∥s∥}^{2}\right)=\int_{f}f^{2}{\left|S(f)\right|}^{2}df$ for a normalized pulse ${∥s∥}^{2}T_{s}=1$ and $\dot{s}$ being the sampled derivative of the pulse with respect to τ. The parameter $\gamma_{\tau }$ is a “whitening gain” for the delay estimation, representing the increased ranging information due to the whitening operation introduced in Equations (11) and (12) (cf., [19]), defined as $\gamma_{\tau }=\beta_{w}^{2}/\beta^{2}$, where $\beta_{w}^{2}$ is the corresponding mean squared bandwidth of the “whitened” pulse, defined as $\beta_{w}^{2}={∥\dot{s}∥}_{H}^{2}/\left(4\pi^{2}{∥s∥}_{H}^{2}\right)$. The factor $\sin^{2}\left(\xi \right)\in \left[0,1\right]$ is a loss factor attributable to the estimation of the nuisance parameter α, arising from the distortion of s through the whitening operation (see Appendix A and [19]), where ξ is the angle between s and $\dot{s}$ in the corresponding Hilbert space H. The factors $\mathrm{SINR}={\left|\alpha \right|}^{2}{∥s∥}_{H}^{2}T_{s}/N_{0}$ and ${\tilde{\mathrm{SINR}}}_{\tau }=\gamma_{\tau }\mathrm{SINR}\sin^{2}\left(\xi \right)$ in Equations (20) and (21) describe the signal-to-interference-plus-noise-ratio (SINR) and the effective SINR for the delay estimation, respectively. For negligible DM, which defaults to the AWGN case, Equation (20) simplifies to the known CRLB for delay estimation [7] (Ch. 3) with *M* independent observations

(22) $$\begin{array}{c} J_{\tau }^{\mathrm{AWGN}}=8\pi^{2}\beta^{2}M\;\mathrm{SNR} \end{array}$$

and signal-to-noise-ratio $\mathrm{SNR}={\left|\alpha \right|}^{2}{∥s∥}^{2}T_{s}/N_{0}$. Introducing the DM, it holds that SINR≤SNR (see Equation (12) and Section 3.4).

### 3.3. Angulation Error Bound (AEB)

In a similar fashion as for the REB, we obtain the angulation error bound (AEB) $A=\sqrt{J_{\phi }^{-1}}$ from Equation (18), which denotes the lower bound of the achievable variance $\mathrm{var}\left\{\hat{\phi }\right\}\geq J_{\phi }^{-1}$ for estimating the AoA of the LoS component. The EFIM element for the angle estimation problem is

(23) $$\begin{array}{cc} J_{\phi } & =8\pi^{2}\left(f_{c}^{2}+\beta^{2}\right)\gamma_{\phi }\mathrm{SINR}\sum_{m=1}^{M}D_{m}^{2}\left(\phi \right) \end{array}$$

(24) $$\begin{array}{c} =8\pi^{2}\left(f_{c}^{2}+\beta^{2}\right){\tilde{\mathrm{SINR}}}_{\phi }\sum_{m=1}^{M}D_{m}^{2}\left(\phi \right). \end{array}$$

In Equation (23), $\gamma_{\phi }=\frac{f_{c}^{2}+\beta_{w}^{2}}{f_{c}^{2}+\beta^{2}}$ is the whitening gain for the AEB, and $D_{m}\left(\phi \right)$ is a factor determined by the array geometry. In Equation (24), the SINR and the whitening gain for the AEB are combined into the effective SINR for the angle estimation ${\tilde{\mathrm{SINR}}}_{\phi }$. The EFIM for the negligible DM case is given by [28]

(25) $$J_{\phi }^{\mathrm{AWGN}}=8\pi^{2}\left(f_{c}^{2}+\beta^{2}\right)\mathrm{SNR}\sum_{m=1}^{M}D_{m}^{2}\left(\phi \right).$$

In contrast to the REB, the AEB can be simplified to obtain a more intuitive expression. The bandwidth term $\beta^{2}$ is negligible due to the usually much higher carrier frequency, i.e., $f_{c}^{2}\gg \beta^{2}$, resulting in $\gamma_{\phi }\approx 1$ and further in ${\tilde{\mathrm{SINR}}}_{\phi }\approx \mathrm{SINR}$ such that we find the AEB to be proportional to

(26) $$\begin{array}{cc} J_{\phi } & =8\pi^{2}\left(f_{c}^{2}+\beta^{2}\right)\;{\tilde{\mathrm{SINR}}}_{\phi }\sum_{m=1}^{M}D_{m}^{2}\left(\phi \right) \end{array}$$

(27) $$\begin{array}{c} \approx 8\pi^{2}f_{c}^{2}\;\mathrm{SINR}\sum_{m=1}^{M}D_{m}^{2}\left(\phi \right) \end{array}$$

(28) $$\begin{array}{c} =8\pi^{2}\;\mathrm{SINR}\sum_{m=1}^{M}\frac{c^{2}}{\lambda^{2}}D_{m}^{2}\left(\phi \right) \end{array}$$

(29) $$\begin{array}{cc} \; & =8\pi^{2}\;\mathrm{SINR}\;M\;D_{\lambda }^{2}\left(\phi \right) \end{array}$$

By setting the carrier frequency in relation to the array geometry, we can define a normalized squared array aperture $D_{\lambda }^{2}\left(\phi \right)$ which captures all the system parameters that influence the AEB. It is defined as

(30) $$\begin{array}{c} D_{\lambda }^{2}\left(\phi \right)=\frac{1}{M}\sum_{m=1}^{M}\frac{d_{m}^{2}}{\lambda^{2}}\sin^{2}\left(\phi -\psi_{m}\right). \end{array}$$

*Geometric interpretation of the AEB:* As becomes obvious when comparing the REB (Equation (20)) with the AEB (Equations (23) and (29)), the latter depends on the direction ϕ. For a uniform linear array (ULA), the CRLB is larger from end-fire direction, i.e., for signals impinging parallel to the array axis, and lower for sources from broadside direction, i.e., perpendicular to the array axis.

*Special case M-ary ULA:* For an *M*-ary ULA with inter-element spacing of λ/2 and resulting $d_{m}=\left(2m-1\right)\lambda /4$, oriented parallel to the *y*-axis, i.e., $\psi_{m}=\pm \pi /2$, Equation (30) is found to be

(31) $$\begin{array}{cc} D_{\lambda }^{2}\left(\phi \right) & =\sum_{m=1}^{M/2}\frac{{\left(2m-1\right)}^{2}}{2M}\sin^{2}\left(\phi -\psi_{m}\right) \end{array}$$

(32) $$\begin{array}{c} =\frac{\sin^{2}\left(\phi +\frac{\pi }{2}\right)}{4M}\sum_{m=1}^{M/2}{\left(2m-1\right)}^{2} \end{array}$$

(33) $$\begin{array}{c} =\frac{\left(M^{2}-1\right)}{24}\frac{1+\cos^{2}\left(2\phi +\pi \right)}{2} \end{array}$$

According to Equation (33), sources from end-fire direction have infinite AEB. As angle estimation is bounded, e.g., for ULAs to $A_{\mathrm{ULA}}\in \left[-\frac{\pi }{2},\frac{\pi }{2}\right]$, the angulation error approaches a uniform distribution on the defined range as the AoA ϕ approaches end-fire direction.

### 3.4. Numerical Evaluation of AEB and REB

#### 3.4.1. Simulation Environment

The theoretical bounds for AEB and REB derived in the previous sections were evaluated numerically and validated using simulations. The evaluation was performed for different pulse bandwidths $1/T_{p}$ of the transmitted signals, where $T_{p}$ is the duration of the transmit pulse. We assumed that the PDP exhibits a double-exponential shape [19,35] with parameters $\gamma_{\mathrm{rise}}=5\;\mathrm{ns}$, $\gamma_{\mathrm{decay}}=20\;\mathrm{ns}$, χ=1, $K_{\mathrm{LoS}}={\left|\alpha \right|}^{2}/\Omega_{1}=0\;\mathrm{dB}$ and SNR=30dB. For simplicity, we used an *M*-ary ULA with inter-element spacing of λ/2 in the simulations, although the theoretical bounds are valid for arbitrary arrays as long as Assumptions (S1)–(S4) are valid. As we linked the inter-element spacing to the carrier frequency $f_{c}$, we kept $f_{c}=7\;\mathrm{GHz}$ fixed.

As transmit pulse s(t), a root-raised-cosine (RRC) pulse was used, as it is often encountered in wireless communications. We set the roll-off factor to $\beta_{\mathrm{roll}-\mathrm{off}}=0.6$ and used the corresponding minimum sampling rate $f_{s}=\left(1+\beta_{\mathrm{roll}-\mathrm{off}}\right)/T_{p}$ needed to avoid aliasing throughout the simulations.

#### 3.4.2. Simulation Results

**Figure 2:**
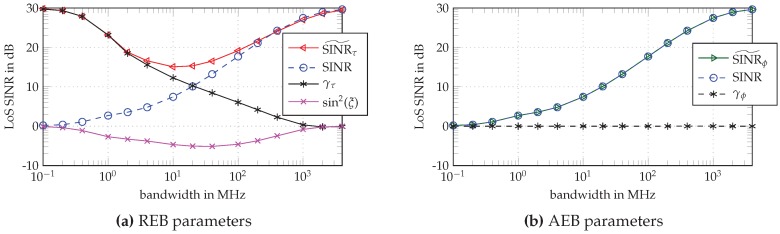
REB and AEB parameters over different pulse bandwidths $1/T_{p}$: effective SINR for the delay estimation ${\tilde{\mathrm{SINR}}}_{\tau }$, effective SINR for the angle estimation ${\tilde{\mathrm{SINR}}}_{\phi }$, SINR, whitening gain for the delay estimation $\gamma_{\tau }$, whitening gain for the angle estimation $\gamma_{\phi }$, and loss factor $\sin^{2}\left(\xi \right)$.

In Figure 2, the EFIM parameters are depicted as a function of the bandwidth according to Equations (20) and (23), separated into parameters for the REB shown in Figure 2a and for the AEB in Figure 2b. In both subfigures, the SINR tends towards the Rician K-factor of the channel at narrow and to the SNR at high bandwidth (BW), which relates to the fading of the LoS signal component in a DM-channel: At high BW, the LoS is separated from the DM such that no fading occurs, while at low BW the complete DM interferes with the LoS leading to a flat fading channel. This implies that the “detectability” of the LoS component is linked to the SINR. The loss factor $\sin^{2}\left(\xi \right)$ observable in the REB and shown in Figure 2a quantifies the SINR that is lost due to estimating the nuisance parameter α in the presence of the non-white, non-stationary DM process $\nu_{m}\left(t\right)$ [19].

The whitening gain $\gamma_{\tau }$ quantifies the SINR gain due to the whitening operation, where the known DM statistics are critical to whiten the observed signal, suppressing the DM. Note that it behaves complementary to the SINR curve (see Figure 2a). The effective SINR for the delay estimation, ${\tilde{\mathrm{SINR}}}_{\tau }$, summarizes these parameters. It shows the same behavior as the SINR for high BW, while, for small BW, it increases towards the SNR, again due to the whitening gain. The effective ${\tilde{\mathrm{SINR}}}_{\tau }$ is thus tied to the distortion effect of the DM. At high and low BW, no pulse distortion occurs as either the DM is separated from the LoS component or it interferes with it completely. However, at intermediate BW, the distortion effect reduces the effective SINR for the delay estimation [19].

For the angle estimation problem (see Figure 2b), no significant whitening gain is possible, as the factor $\gamma_{\phi }=\frac{f_{c}^{2}+\gamma_{\tau }\beta^{2}}{f_{c}^{2}+\beta^{2}}$ does not substantially exceed 1. Remember that $\beta /f_{c}\ll 1$ at typical carrier frequencies of radio signals. Furthermore, at typical carrier frequencies, if the fractional BW is high, also the absolute BW is high, meaning that the factor $\gamma_{\tau }$ is small. Thus, the whitening factor $\gamma_{\phi }$ is approximately 1, which in turn ties the effective ${\tilde{\mathrm{SINR}}}_{\phi }$ for the angle estimation to the SINR, as assumed in Equation (29).

**Figure 3:**
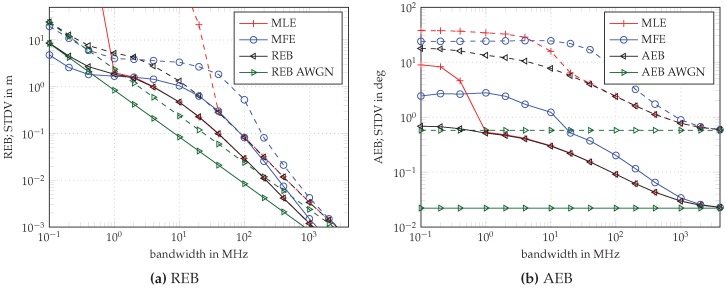
Ranging error bound (REB) and angulation error bound (AEB) over different pulse bandwidths $1/T_{p}$, and performance results for matched filter estimator (MFE) and maximum likelihood estimator (MLE). Simulations were performed using $N_{r}=10,000$ realizations for ULAs with M=2 (dashed lines) and M=16 (solid lines) elements and inter-element spacing of λ/2; the resulting array aperture is (M−1)λ/2.

The results for the REB and the AEB are shown in Figure 3 for ULAs with M=2 and M=16 elements (dashed and solid, respectively), spaced by λ/2. The CRLB for AWGN-only in green and AWGN-plus-DM in black is compared to the standard deviation achieved by two types of estimators: results for a matched filter estimator (MFE) are indicated in blue and for a maximum likelihood estimator (MLE) in red.

In accordance with the results in [19], the MFE starts to deviate from the theoretical bound below a BW of 400 MHz, whereas the MLE follows the bound down to 40MHz for M=2 and down to 1MHz for M = 16 (see Figure 3a). The MLE follows the bound longer due to the whitening operation which assumes known AWGN and DM statistics. For an increasing number of antenna elements *M*, the MLE follows the REB much longer due to the diversity gain, i.e., due to the M-fold increase of the SINR by performing multiple measurements with *M* antenna array elements. The REB improves correspondingly with $1/\sqrt{M}$.

In the case of lower BW, the MFE again converges to the bound as the MFE starts to make use of the DM and LoS power. It even outperforms the REB, which is somewhat pessimistic at low bandwidth due to the neglected trace-term in the derivation of the FIM (see Appendix A). For a numerical evaluation of this effect, see [19]. The whitening operation of the MLE on the other hand reduces the SNR [19] at low BW, as already discussed.

The effect of the DM on the AEB is different to that on the REB: the DM causes the AEB to deviate from the AWGN bound below 1 GHz, but the whitening operation does not influence the AEB. The estimators perform similarly (see Figure 3b): The MFE leaves the bound at higher BW and returns to it at lower BW. The MLE follows the AEB down to 40MHz and 1MHz corresponding to the detectability of the LoS component within the noise, after the whitening. Again, the diversity gain is evident by the improved convergence for M=16 in comparison with M=2. The improvement of AoA accuracy, when comparing the results for M=2 and M=16, is attributed to the normalized squared array aperture $D_{\lambda }^{2}\left(\phi \right)$ as shown in Equation (33), which directly depends on the total number *M* of λ/2-spaced array elements.

An important observation from the theoretical results is the fact that the performance of both (ToA and AoA) estimators is limited by the influence of DM. Increasing the SNR of the radio signal will not improve the performance, while antenna diversity will help. Furthermore, we would like to emphasize the influence of the DM on the AEB, especially at smaller bandwidths. While the AWGN AEB does not depend on the bandwidth, we show that, by considering the influence of the DM, the AEB depends on the bandwidth (see Figure 3b).

## 4. Cramér–Rao Lower Bound for Positioning

Building on the REB and AEB derived in the previous sections, we join the two bounds to gain insight into the position error bound (PEB) which gives the achievable accuracy for estimating the agent position $p={\left[\;x,y\;\right]}^{T}$ considering one or multiple fixed anchors at positions $q^{\left(\ell \right)}$ equipped with antenna arrays. We therefore reintroduce the anchor index *ℓ* at the proper quantities.

For simplicity of notation, we assume that each anchor is equipped with identical arrays and performs its measurements with the agent in a similar fashion, e.g., with the same bandwidth β and carrier frequency $f_{c}$, which is a reasonable assumption for most communication systems.

### 4.1. Position Error Bound (PEB)

From the FIM for the parameter vector $\psi^{\left(\ell \right)}={\left[\;x,y,R\alpha^{\left(\ell \right)},I\alpha^{\left(\ell \right)}\;\right]}^{T}$, containing the agent position and the LoS amplitudes at each anchor *ℓ*, we acquire the corresponding single-anchor CRLB as

(34) $$\begin{array}{c} E_{\psi }\;\left\{\left({\hat{\psi }}^{\left(\ell \right)}-\psi^{\left(\ell \right)}\right){\left({\hat{\psi }}^{\left(\ell \right)}-\psi^{\left(\ell \right)}\right)}^{T}\right\}⪰{J_{\psi }^{\left(\ell \right)}}^{-1}. \end{array}$$

The FIM for the parameter vector $\psi^{\left(\ell \right)}$ can be computed from the FIM for the AoA and ToA as

(35) $$\begin{array}{c} J_{\psi }^{\left(\ell \right)}=T^{\left(\ell \right)}J_{\theta }^{\left(\ell \right)}{T^{\left(\ell \right)}}^{T} \end{array}$$

with $T^{\left(\ell \right)}$ denoting the Jacobian matrix containing the partial derivatives of the parameter vector $\psi^{\left(\ell \right)}$ with respect to the parameters in $\theta^{\left(\ell \right)}$ [20,24,25]

(36) $$\begin{array}{cc} T^{\left(\ell \right)} & =\frac{\partial {\theta^{\left(\ell \right)}}^{T}}{\partial \psi^{\left(\ell \right)}}=\left[\begin{array}{cc} P^{\left(\ell \right)} & 0\\ 0 & I_{2\times 2} \end{array}\right]\in R^{4\times 4} \end{array}$$

with

(37) $$\begin{array}{cc} P^{\left(\ell \right)} & =\left[\begin{array}{cc} \frac{\partial \tau^{\left(\ell \right)}}{\partial x} & \frac{\partial \phi^{\left(\ell \right)}}{\partial x}\\ \frac{\partial \tau^{\left(\ell \right)}}{\partial y} & \frac{\partial \phi^{\left(\ell \right)}}{\partial y} \end{array}\right]=\left[\begin{array}{cc} \frac{\cos\phi^{\left(\ell \right)}}{c} & \frac{\sin\phi^{\left(\ell \right)}}{\tau^{\left(\ell \right)}c}\\ \frac{\sin\phi^{\left(\ell \right)}}{c} & -\frac{\cos\phi^{\left(\ell \right)}}{\tau^{\left(\ell \right)}c} \end{array}\right]\in R^{2\times 2} \end{array}$$

Each anchor *ℓ*—having a line-of-sight connection to the agent—provides position information about the agents position p by performing independent measurements. Thus, we define the PEB via the EFIM for the position utilizing all anchors from the set of LoS anchors $\ell \in L_{0}$ as

(38) $$\begin{array}{c} P=\sqrt{\mathrm{tr}\left\{J_{P}^{-1}\right\}}=\sqrt{\mathrm{tr}\left\{{\left[J_{\psi }\right]}_{2\times 2}^{-1}\right\}}. \end{array}$$

where the EFIM for the position $J_{P}$ is obtained by

(39) $$\begin{array}{c} J_{P}=\sum_{\ell \in L_{0}}{\left[J_{\psi }^{\left(\ell \right)}\right]}_{2\times 2}=\sum_{\ell \in L_{0}}P^{\left(\ell \right)}{\left[J_{\theta }\right]}_{2\times 2}P^{T}=\sum_{\ell \in L_{0}}P^{\left(\ell \right)}\left(A^{\left(\ell \right)}-B^{\left(\ell \right)}{D^{\left(\ell \right)}}^{-1}{B^{\left(\ell \right)}}^{T}\right){P^{\left(\ell \right)}}^{T} \end{array}$$

The matrices $A^{\left(\ell \right)}$, $B^{\left(\ell \right)}$ and $D^{\left(\ell \right)}$ are found using Equations (15)–(17) for each anchor *ℓ*. By further inserting Equation (18) and the corresponding EFIMs for the AoAs and ToAs from Equations (21) and (29), we obtain the multiple anchor EFIM for the position as

(40) $$\begin{array}{cc} J_{P}=J_{P,\phi }+J_{P,\tau } & =\sum_{\ell \in L_{0}}\frac{J_{\phi }^{\left(\ell \right)}}{{d^{\left(\ell \right)}}^{2}}\;J_{r}(\phi^{\left(\ell \right)}+\frac{\pi }{2})+\frac{J_{\tau }^{\left(\ell \right)}}{c^{2}}\;J_{r}\left(\phi_{\ell }\right) \end{array}$$

(41) $$\begin{array}{c} \approx \sum_{\ell \in L_{0}}\frac{8\pi^{2}}{{d^{\left(\ell \right)}}^{2}}\;D_{\lambda }^{2}\left(\phi^{\left(\ell \right)}\right)\;M\;{\mathrm{SINR}}^{\left(\ell \right)}\;J_{r}\left(\phi_{\ell }+\frac{\pi }{2}\right)+\frac{8\pi^{2}}{c^{2}}\beta^{2}\;M\;{\tilde{\mathrm{SINR}}}_{\tau }^{\left(\ell \right)}\;J_{r}\left(\phi_{\ell }\right) \end{array}$$

The matrices $J_{P,\phi }$ and $J_{P,\tau }$ are the position EFIMs obtained from AoAs and ToAs, respectively. They also show the available information for either AoA-only or ToA-only positioning. The matrix $J_{r}\left(\phi \right)$ indicates the direction from which the information arrives at the agent and is usually called ranging direction matrix [8]. It arises from the Jacobian transforming the FIM (or EFIM) for ToA and AoA to the position and has the form

(42) $$\begin{array}{c} J_{r}\left(\phi^{\left(\ell \right)}\right)=\left[\begin{array}{cc} \cos^{2}\phi^{\left(\ell \right)} & \sin\phi^{\left(\ell \right)}\cos\phi^{\left(\ell \right)}\\ \sin\phi^{\left(\ell \right)}\cos\phi^{\left(\ell \right)} & \sin^{2}\phi^{\left(\ell \right)} \end{array}\right]=e\left(\phi^{\left(\ell \right)}\right)e^{T}\left(\phi^{\left(\ell \right)}\right) \end{array}$$

where $e\left(\phi^{\left(\ell \right)}\right)={\left[\;\cos\phi^{\left(\ell \right)},\sin\phi^{\left(\ell \right)}\;\right]}^{T}$ is a unit vector pointing in direction of the agent $\phi^{\left(\ell \right)}$, which is also the sole eigenvector of the ranging direction matrix. This formulation clearly shows that both range-only and angle-only positioning are ill-posed problems for the case of a single LoS anchor, as the EFIM for the position will be rank deficient and thus not invertible.

**Figure 4:**
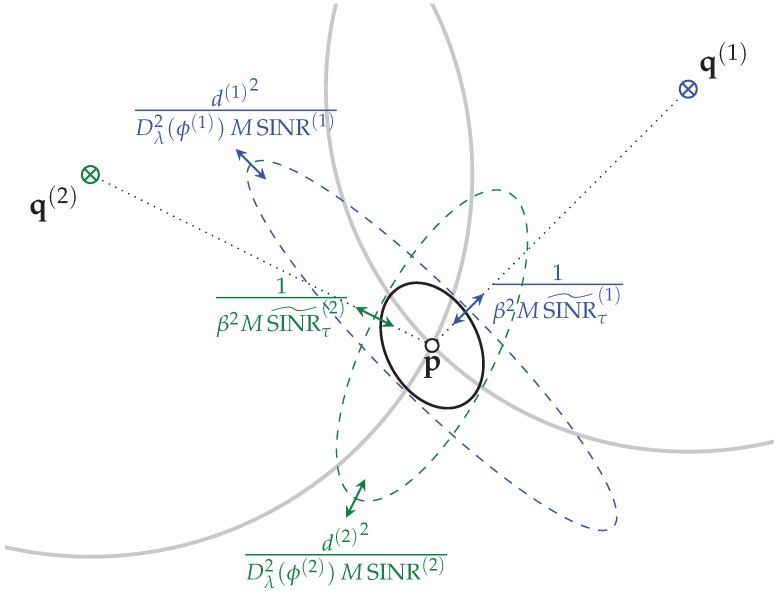
PEB as stated in Equation (41) for two anchors $q^{\left(1\right)}$ and $q^{\left(2\right)}$ equipped with antenna arrays, visualizing the influence of the different parts attributable to ranging (in direction of the agent) and angulation (perpendicular to the agent direction) that contribute to the positioning accuracy of an agent p.

*Geometric interpretation of the PEB:* The EFIM in Equation (41) further supports the well established result that ranging provides position information in direction of the anchor, whereas angulation is responsible for information perpendicular to the anchor direction (see e.g., [24]). A graphical interpretation illustrating Equation (41) is shown in Figure 4 where we showcase the most important parameters that influence the achievable positioning accuracy. As discussed in Section 3.4, the angulation accuracy is inversely proportional to the SINR, which in turn depends on the signal bandwidth β. The achievable ranging accuracy depends on the bandwidth as well, thus the PEB in a DM channel scales with the bandwidth both in direction and perpendicular to the agent.

### 4.2. Position Error Bound (PEB) Considering Path-Overlap

The previously described signal model included only a single specular MPC, namely the LoS. Especially for signals of high bandwidth or in indoor scenarios, the assumption of a single specular component is no longer viable. Thus, we adapt the signal model to include multiple specular components k∈{1,⋯,K} with ToAs $\tau_{k}$, AoAs $\phi_{k}$ and complex-amplitudes $\alpha_{k}$. This enables us to examine the influence of path-overlap (see, e.g., [18]) between the LoS component and later arriving specular MPCs in dense multipath channels.

For each anchor (we again omit the index *ℓ* in the following equations for readability), we get the extended signal model as

(43) $$\begin{array}{cc} r_{m}\left(t\right) & =\sum_{k=1}^{K}a_{mk}s\left(t-\tau_{mk}\right)+\left(s*\nu_{m}\right)\left(t\right)+w_{m}\left(t\right), \end{array}$$

where we again assume that Equation (3) and (S1) hold.

In comparison to the LOS only signal model, see Equation (4), we redefine the auto-correlation function of the DM process as

(44) $$\begin{array}{cc} E_{\nu }\;\left\{\nu_{m}\left(t\right){\left[\nu_{m^{\prime }}\left(u\right)\right]}^{*}\right\} & \approx S_{\nu }\left(t-\tau_{m1}\right)\delta \;\left(t-u\right)\delta \left[m-m^{\prime }\right]. \end{array}$$

where the onset of the DM process is the ToA of the LoS signal $\tau_{m1}$.

Without any information about the room geometry, we can still only use the LoS signal for positioning. The parameters of all other specular components are regarded as nuisance parameters in the estimation process; the extended parameter vector is thus $\theta^{\prime }={\left[\;\phi_{1},\cdots ,\phi_{K},\tau_{1},\cdots ,\tau_{K},R\alpha_{1},\cdots ,R\alpha_{K},I\alpha_{1},\cdots ,I\alpha_{K}\;\right]}^{T}$. The previous definition of the overall covariance matrix $C_{m}$ of the sampled noise processes at each element *m* is still valid, thus the likelihood function for $r_{m}$ is

(45) $$\begin{array}{cc} p\;\left(r_{m};\theta^{\prime }\right) & \propto \exp\;\{-{\left(r_{m}-S_{m}a_{m}\right)}^{H}C_{m}^{-1}\left(r_{m}-S_{m}a_{m}\right)\} \end{array}$$

with $S_{m}=\left[\;s_{m1},\cdots ,s_{mk},\cdots ,s_{mK}\right]$, $s_{mk}={\left[\;s\left(-\tau_{mk}\right),s\left(T_{s}-\tau_{mk}\right),\cdots ,s\left(\left(N-1\right)T_{s}-\tau_{mk}\right)\right]}^{T}$ and $a_{m}=\left[a_{m1},\cdots ,a_{mk},\cdots ,a_{mK}\right]$. Noise and DM are still assumed uncorrelated at each element *m* (S3), therefore the likelihood function $p\;\left(r;\theta \right)$ for the stacked observation vector r is again factorized as $p\;\left(r;\theta \right)=\prod_{m=1}^{M}p\;\left(r_{m};\theta \right)$. Using the definition of the FIM (Equation (7)), we find $J_{\theta^{\prime }}$

(46) $$\begin{array}{c} J_{\theta^{\prime }}=\sum_{m=1}^{M}J_{\theta^{\prime },m}=\left[\begin{array}{cc} A & B\\ B^{T} & D \end{array}\right]\in R^{4K\times 4K} \end{array}$$

with the matrix blocks

(47) $$\begin{array}{cc} A & =\left[\begin{array}{cc} J_{\phi \phi } & J_{\phi \tau }\\ J_{\tau \phi } & J_{\tau \tau } \end{array}\right] \end{array}$$

(48) $$\begin{array}{cc} B & =\left[\begin{array}{cc} J_{\phi R\alpha } & J_{\phi I\alpha }\\ J_{\tau R\alpha } & J_{\tau I\alpha } \end{array}\right]=\left[\begin{array}{c} B_{\phi }\\ B_{\tau } \end{array}\right] \end{array}$$

(49) $$\begin{array}{cc} D & =\left[\begin{array}{cc} J_{R\alpha R\alpha } & 0\\ 0 & J_{I\alpha I\alpha } \end{array}\right]. \end{array}$$

The elements of the matrix blocks in Equations (47)–(49) are described in detail in Appendix B. By examining the elements of the matrix blocks in Equation (46), we find that path-overlap occurs if the Fourier-weighted inner product between two specular MPCs *k* and κ is non-zero, i.e., the terms ${\langle s_{mk},s_{m\kappa }\rangle }_{H_{m}}\neq 0$ in $J_{\theta^{\prime }}$.

To find the FIM for the parameter vector $\psi^{\prime }={\left[\;x,y,R\alpha_{1},\cdots ,R\alpha_{K},I\alpha_{1},\cdots ,I\alpha_{K}\;\right]}^{T}$, we introduce the Jacobian matrix T as

(50) $$\begin{array}{c} T=\frac{\partial {\theta^{\prime }}^{T}}{\partial \psi^{\prime }}=\left[\begin{array}{cc} P & 0\\ 0 & I_{2K\times 2K} \end{array}\right]\in R^{2+2K\times 4K} \end{array}$$

with the sub-block

(51) $$\begin{array}{c} P=\left[\begin{array}{cc} \frac{\partial \phi^{T}}{\partial x} & \frac{\partial \tau^{T}}{\partial x}\\ \frac{\partial \phi^{T}}{\partial y} & \frac{\partial \tau^{T}}{\partial y} \end{array}\right]=\left[\begin{array}{cccccccc} \frac{\partial \phi_{1}}{\partial x} & 0 & \cdots & 0 & \frac{\partial \tau_{1}}{\partial x} & 0 & \cdots & 0\\ \frac{\partial \phi_{1}}{\partial y} & 0 & \cdots & 0 & \frac{\partial \tau_{1}}{\partial y} & 0 & \cdots & 0 \end{array}\right]\in R^{2\times 2K} \end{array}$$

Finally, to derive the EFIM for positioning using multiple anchors, we re-introduce the anchor index *ℓ* at all relevant quantities. With the assumption of independent measurements obtained between each anchor *ℓ* and the agent, we get the EFIM as

(52) $$\begin{array}{cc} J_{P}=\sum_{\ell \in L_{0}}{\left[J_{\psi^{\prime }}^{\left(\ell \right)}\right]}_{2\times 2} & =\sum_{\ell \in L_{0}}P^{\left(\ell \right)}{\left[J_{\theta^{\prime }}^{\left(\ell \right)}\right]}_{2\times 2}{P^{\left(\ell \right)}}^{T}=\sum_{\ell \in L_{0}}P^{\left(\ell \right)}\left(A^{\left(\ell \right)}-B^{\left(\ell \right)}{D^{\left(\ell \right)}}^{-1}{B^{\left(\ell \right)}}^{T}\right){P^{\left(\ell \right)}}^{T} \end{array}$$

from which we obtain the PEB using Equation (38).

*Comparison to single-component PEB:* For decreasing path-overlap, the matrices $B_{\phi }$ in Equation (48) as well as $J_{\tau \phi }$ and $J_{\phi \tau }$ in Equation (47) will approach zero due to ${\langle s_{mk},s_{m\kappa }\rangle }_{H_{m}}\approx 0\;\forall \;m$ for k≠κ, and in combination with the chosen reference point (e.g., see Appendix A). Furthermore, the matrices $J_{R\alpha R\alpha }$ and $J_{I\alpha I\alpha }$ in Equation (49) and $J_{\tau R\alpha }$ and $J_{\tau I\alpha }$ in Equation (48) will become diagonal due to the same mechanism and we will experience subtractive terms leading to the factor $\sin^{2}\left(\xi \right)$, which allow formulation of the effective SINR ${\tilde{\mathrm{SINR}}}_{\tau }$ for the LoS component. As we can only use the LoS signal component for positioning, the PEB in Equation (52) will become Equation (41). In contrast, if path-overlap occurs, the subtractive term becomes none-zero, corresponding to a loss of useful position information. The resulting PEB can still be evaluated numerically as demonstrated in the following section.

### 4.3. Numerical Evaluation of the PEB

#### 4.3.1. Simulation Environment

**Figure 5:**
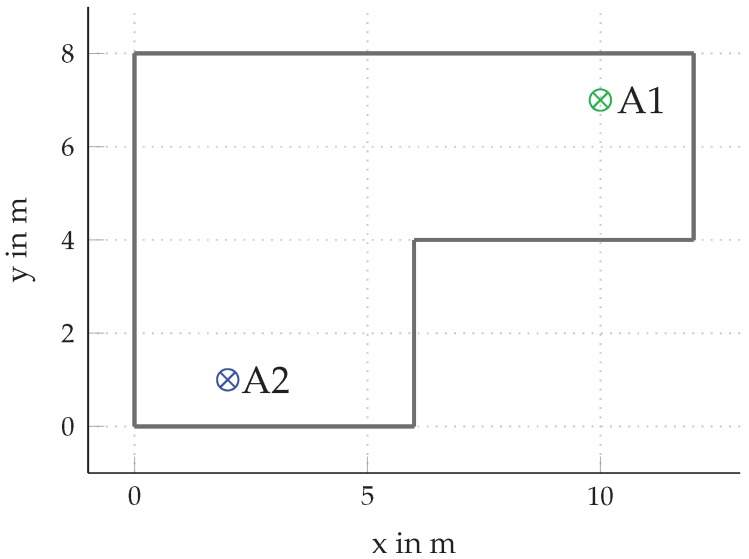
Floorplan of the synthetic environment used for the PEB evaluation with two anchors A1 (⊗) and A2 (⊗) at positions $q^{\left(1\right)}={\left[10,7\right]}^{T}$ and $q^{\left(2\right)}={\left[2,1\right]}^{T}$. The specular components were modeled via reflections at the walls. We modeled reflections up to second order, i.e., two wall interactions.

To evaluate the different formulations of the PEB obtained in the previous section, we performed numerical evaluations in a synthetic indoor environment where we modeled specular reflections up to second order. An overview of the floorplan of the room is shown in Figure 5, where the positions $q^{\left(1\right)}={\left[10,7\right]}^{T}$ and $q^{\left(2\right)}={\left[2,1\right]}^{T}$ of the two anchors used for simulations are labeled as A1 (⊗) and A2 (⊗). Each anchor is equipped with a 2×2 uniform rectangular array (URA) oriented parallel to the x- and y-axes with identical element spacings of λ/2 in x- and y-directions. We examine the achievable positioning accuracy over the whole room. The transmitting agent, equipped with a single antenna, is placed on grid-points within the room spaced by 2cm in x- and y-directions. At each grid-point, the different variants of the PEB are evaluated.

In accordance with the examination of REB and AEB, we assumed that the statistical properties of the DM can be estimated from a number of snapshots taken at the same position and are thus assumed to be known. The DM parameters used are identical to the ones used in Section 3.4.1, i.e., we set $\gamma_{\mathrm{rise}}=5\;\mathrm{ns}$, $\gamma_{\mathrm{decay}}=20\;\mathrm{ns}$ and χ=1. These were kept constant over the room to allow a qualitative evaluation of the PEB. (The authors are aware that this is by no means a realistic assumption as the position of the agent within the environment might influence the DM statistics. It is considered beyond the scope of this work to formulate a realistic simulation model for the behavior of the DM across a room.) To model the amplitudes of the specular components, we assumed a pathloss coefficient of η=1.6 characterizing an indoor LoS environment [36] and assumed that each reflection at a wall results in a further reduction of 3dB. We computed the amplitude of each MPC relative to the LoS amplitude at a distance of 1m, which we set to $\alpha_{1\;m}=1$ without loss of generality. The Rician K-factor $K_{\mathrm{LoS}}$ of the LoS power with respect to the mean power of the DM was again set to $K_{\mathrm{LoS}}={\left|\alpha_{1\;m}\right|}^{2}/\Omega_{1}=0\;\mathrm{dB}$, also defined at a distance of 1m. The SNR was kept constant over the whole room at SNR=29.5dB. The used transmit pulse shape is the same as in Section 3.4.1, i.e., a RRC-pulse with $\beta_{\mathrm{roll}-\mathrm{off}}=0.6$ and a sampling rate $f_{s}=\left(1+\beta_{\mathrm{roll}-\mathrm{off}}\right)/T_{p}$.

#### 4.3.2. Simulation Results

**Figure 6:**
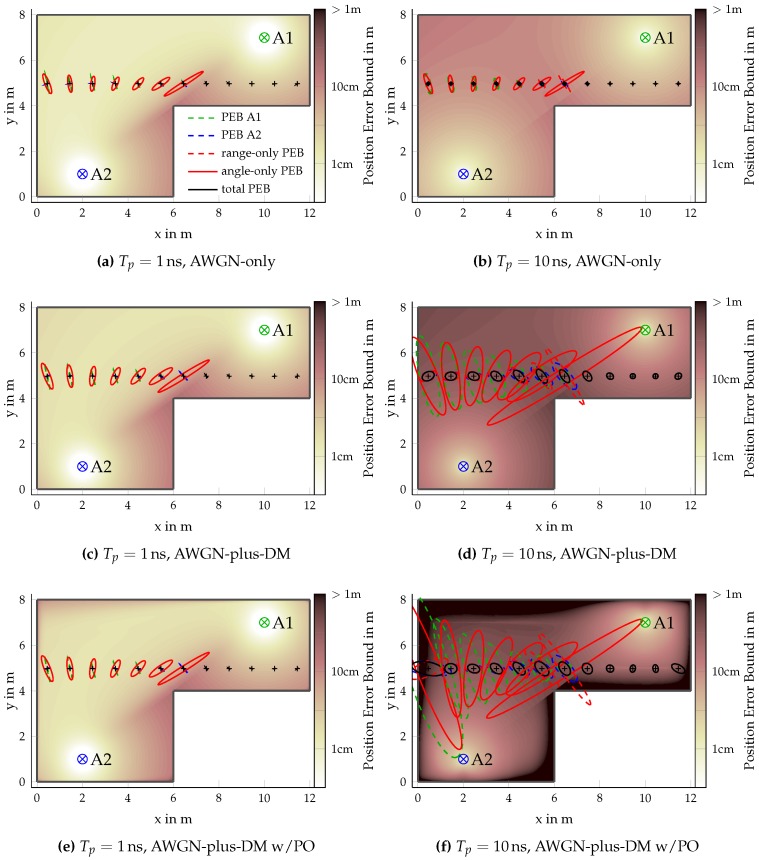
PEB ellipses (1-fold) for LoS-only positioning using a 2×2 array at anchors A1 (⊗) and A2 (⊗) for pulse durations $T_{p}=\left\{1,10\right\}\;\mathrm{ns}$. Ellipses for range- and angle-only positioning are only shown where two anchors are visible. Underlying colors indicate the total PEB.

We started by evaluating the PEB over the room for different channels: (i) AWGN-only (The AWGN-only channel, meaning that no DM is simulated, is used as a baseline as it has been evaluated in [22,23,24].); (ii) AWGN-plus-DM (cf. Section 4.1); and (iii) AWGN-plus-DM with path-overlap (cf. Section 4.2). Figure 6 shows the PEB over the room alongside the PEB ellipses for the different types of positioning, namely range-only and angle-only as well as the contributions of each anchor individually (Equation (40)) and the resulting full PEB (Equations (41) and (52)).

To examine the bandwidth dependence of the PEB, we performed the simulations at bandwidths of 1GHz and 100MHz shown in the left and right columns of Figure 6, respectively. Starting with the case of AWGN-only in Figure 6a,b, we observed a rather homogeneous distribution of the PEB in regions where both anchors are visible. When the agent and the anchors become increasingly co-linear, the accuracy degrades as expected. Furthermore, the well known distance dependent degradation is seen. This is explained by the lower accuracy obtained from the AoA, as its accuracy is inversely proportional to the distance, see, e.g., Equations (41) and (52) and Figure 4. Comparing the PEB for different bandwidths of 1GHz (see Figure 6a) and 100MHz (Figure 6b), we see that the range induced accuracy depends on the bandwidth and not on the distance, while the contribution of the AoA is independent of the bandwidth but in turn decreases with distance.

Comparing the cases of AWGN-only (Figure 6a,b) and AWGN-plus-DM (Figure 6c,d), the negative effect of the DM is clearly visible as SINR<SNR (see Figure 2). The PEB of the AWGN-plus-DM case will thus be larger than for the AWGN-only case. The SINR is bandwidth dependent, hence, the influence of the DM is stronger at smaller bandwidth. This was already shown in Figure 3 for the AEB and the REB, which are in turn tightly related to the PEB, e.g., Equation (41).

It is interesting to note that, in contrast to the AWGN-only case, angle estimation becomes bandwidth dependent in DM. This can be seen clearly by comparing the angle-only PEB for the different bandwidths between AWGN-only and AWGN-plus-DM. For the AWGN-only case, the AoA-only PEB is worse than the REB-only PEB, because it is dependent on the LoS signal component by direction and distance. In the AWGN-plus-DM case, this effect is even more severe, because the angle does not profit from the whitening operation, thus, ${\tilde{\mathrm{SINR}}}_{\phi }=\mathrm{SINR}\leq {\tilde{\mathrm{SINR}}}_{\tau }$.

The influence of specular components overlapping with the LoS signal component is shown in Figure 6e,f, which evaluates the PEB from Equation (52). At the high bandwidth of 1GHz, the influence is negligible over most of the room, due to the short pulse duration of 1ns≈0.3m. It is only slightly visible in room corners. The effect of path-overlap is much more severe when we reduced the bandwidth to 100MHz. Due to the now much longer pulses, overlap occurs in regions near the walls, resulting in higher PEB.

These result show that especially in the lower bandwidth region below 1GHz the AWGN-only assumption results in an overly optimistic PEB, as it neglects both the influences of the DM as well as the possibility of path-overlap caused by specular components. As we usually see specular components apart from the LoS in indoor scenarios, these results again highlight the difficulties due to DM and path-overlap.

#### 4.3.3. Single-Anchor Positioning

**Figure 7:**
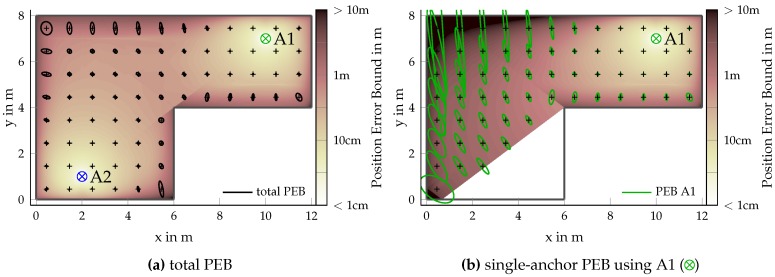
PEB ellipses (1-fold) for AWGN-plus-DM with path-overlap and $T_{p}=10\;\mathrm{ns}$ comparing the full PEB using anchors A1 (⊗) and A2 (⊗) and single anchor positioning using solely A1 (⊗). Underlying colors show the same PEB as the ellipses.

Figure 7 examines the contribution of a single anchor in more detail by comparing the PEB for a single-anchor setup using only A1 (see Figure 7b) and comparing it to the full PEB when using both anchors (Figure 7a). The case of AWGN-plus-DM with path-overlap was considered for a bandwidth of 100MHz. The trivial result, when using only a single anchor, is that, at non-LoS locations, no position can be obtained. From the orientation of the PEB ellipses, we again observed the decreasing accuracy perpendicular to the anchor direction due to the angulation accuracy being inversely proportional to the distance. It should further be noted that both angle and range estimation are influenced by path-overlap, resulting in general in a location-dependent deterioration of the PEB, most severely in room corners. The distance deterioration is rather well compensated by adding a second anchor.

#### 4.3.4. Range-Only and Angle-Only Positioning

**Figure 8:**
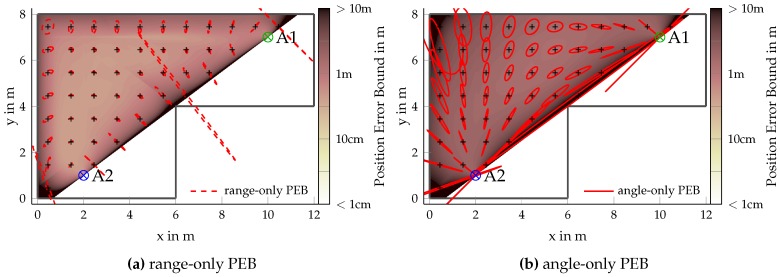
PEB ellipses (1/4-fold) for AWGN-plus-DM with path-overlap and $T_{p}=10\;\mathrm{ns}$ comparing angle-only and range-only positioning (both anchors are needed). Underlying colors show the same PEB as the ellipses.

Figure 8 shows the PEB when assuming that both anchors can either perform only range measurements (Figure 8a, performing multilateration) or only angle measurements (Figure 8b, performing multiangulation), again assuming an AWGN-plus-DM channel with path-overlap for a bandwidth of $1/T_{p}=100\;\mathrm{MHz}$. As a minimum of L=2 anchors providing LoS signals are needed, positioning is not possible over the whole room, as indicated by the underlayed color plots. Comparing the error ellipses in both plots shows that range and angle information are perpendicular to one another. We again observe that multiangulation suffers from the increasing distance and path-overlap, whereas multilateration is independent of the distance between anchor and agent but still subject to path-overlap. Even thoguh the position related information contained in angle and range measurements are perpendicular, both cases suffer greatly from co-linearity of anchors and agent. Joint positioning using angles and ranges can nevertheless obtain high accuracy as shown in Figure 7a.

## 5. Conclusions

The ranging and angulation error bounds (REB and AEB, respectively) were evaluated for time-of-arrival (ToA) and angle-of-arrival (AoA) estimation in dense multipath channels, examining the influence of the number and geometry of array elements as well as the effect of the bandwidth of the transmitted signal. Simulation results are presented for a matched filter estimator (MFE) and a maximum likelihood estimator (MLE), validating these results. Furthermore, the positioning performance was analyzed, considering these two measurement types, multiple anchors, and the influence of path-overlap by specular multipath components (MPCs).

The multipath signal model makes our numerical results significantly more realistic than previous analytical results. Most notably, it is shown that, due to the impact of dense multipath, the achievable accuracy for AoA estimation becomes bandwidth dependent. This is a novel finding and stands in contrast to the known results for the AWGN-only channel, where the AoA accuracy appears to be widely independent of the employed signal bandwidth. Specifically, the AEB scales with the squared array aperture, the number of array-antenna elements, and the signal-to-interference-plus-noise ratio (SINR). The REB scales with the squared signal bandwidth, the number of antennas, and the SINR. The SINR itself quantifies the influence of the dense multipath. It also scales with the bandwidth, because the interfering multipath can be better resolved at a higher signal bandwidth. The relevance of our work lies in the exact numerical quantification of these different influencing factors, which allows for the evaluation of trade-offs between various system configurations under realistic channel conditions.

The analysis of the position error bound (PEB) illustrates the relationship between the ToA and AoA information in multi-anchor positioning scenarios and the influence of the anchor–agent placement. The different measurement types can complement one another. The AoA will be most useful at close range—allowing for accurate single-anchor positioning at close range—while, at far range, only the ranging information remains accurate. Interfering specular multipath components have a maximum detrimental influence near the walls.

Future work may couple the presented theoretical framework with a detailed, parameterized channel model of the application environment to study the expected, site-specific positioning performance as a function of a wide range of system parameters.

## Abbreviations

AEB	Angulation Error Bound
AoA	Angle of Arrival
AWGN	Additive White Gaussian Noise
BW	Bandwidth
CRLB	Cramér–Rao Lower Bound
DM	Dense Multipath
EFIM	Equivalent FIM
FIM	Fisher Information Matrix
LoS	Line-of-Sight
MFE	Matched Filter Estimator
MLE	Maximum Likelihood Estimator
MPC	Multipath Component
TDoA	Time Difference of Arrival
ToA	Time of Arrival
PDP	Power Delay Profile
PEB	Position Error Bound
REB	Ranging Error Bound
RSS	Received Signal Strength
SINR	Signal to Interference-plus-Noise Ratio
ULA	Uniform Linear Array
URA	Uniform Rectangular Array
US	Uncorrelated Scattering
UWB	Ultra-Wide-Bandwidth

## Appendix A. Fisher Information for ToA and AoA (LoS-only)

This appendix gives a more detailed view on the elements of the FIM $J_{\theta ,m}$ as given in Equation (14) for a parameter vector θ, i.e., the case of a single line of sight in dense multipath. We obtained each FIM element from [7] (Sec. 15.7)

Jθkθκ,m=2Re∂μmH∂θkCm−1∂μm∂θκ+trCm−1∂Cm∂θkCm−1∂Cm∂θκ

where $\mu_{m}=a_{m}s_{m}$ and $\theta_{k}$ is the *k*th element of the parameter vector θ. For simplicity, we assume that the trace term $\mathrm{tr}\left\{\cdot \right\}$ only has negligible influence [19]. Due to the symmetry of the FIM, it holds that $J_{\theta_{k}\theta_{\kappa },m}=J_{\theta_{\kappa }\theta_{k},m}$. Inserting the likelihood function (Equation (10)) into Equation (7) and using the fact that $|a_{m}{|}^{2}$, ${∥s_{m}∥}_{H_{m}}^{2}$, ${∥{\dot{s}}_{m}∥}_{H_{m}}^{2}$ and ${\langle s_{m},{\dot{s}}_{m}\rangle }_{H_{m}}$ are all equal for different *m*, we drop the index *m*. The FIM elements on the main-diagonal are computed as

$$\begin{array}{cc} J_{\phi \phi ,m} & ={2|\alpha |}^{2}D_{m}^{2}\left(\phi \right)\left({\left(2\pi f_{c}\right)}^{2}{∥s∥}_{H}^{2}+{∥\dot{s}∥}_{H}^{2}\right)\\ J_{\tau \tau ,m} & ={2|\alpha |}^{2}\left({\left(2\pi f_{c}\right)}^{2}{∥s∥}_{H}^{2}+{∥\dot{s}∥}_{H}^{2}\right)\\ J_{R\alpha R\alpha ,m} & =J_{I\alpha I\alpha ,m}=2{∥s∥}_{H}^{2}. \end{array}$$

The off-diagonal FIM elements are

Jϕτ,m=2|α|2Dm(ϕ)(2πfc)2sH2+s˙H2JϕRα,m=2Dm(ϕ)2πfcImαsH2+Reαs,s˙HJϕIα,m=2Dm(ϕ)−2πfcReαsH2+Imαs,s˙HJτRα,m=22πfcImαsH2+Reαs,s˙HJτIα,m=2−2πfcReαsH2+Imαs,s˙HJRαIα,m=0.

The influence of the array geometry is captured in $D_{m}\left(\phi \right)$ as

(A1) $$\begin{array}{c} D_{m}\left(\phi \right)=\frac{\partial \tau_{m}}{\partial \phi }=\frac{d_{m}}{c}\sin\left(\phi -\psi_{m}\right). \end{array}$$

By choosing the reference point as the center of mass of the array, the sum over *m* for the off-diagonal elements containing $D_{m}\left(\phi \right)$ implies $\sum_{m}D_{m}\left(\phi \right)=0$, hence resulting in $\sum_{m}J_{\phi \tau ,m}=\sum_{m}J_{\phi R\alpha ,m}=\sum_{m}J_{\phi I\alpha ,m}=0$.

The loss factor, due to estimating the amplitudes α, arises as a direct consequence of the subtractive term in Equation (18) and is

(A2) $$\begin{array}{cc} \sin^{2}\left(\xi \right) & =1-\frac{|{\langle s,\dot{s}\rangle }_{H}{|}^{2}}{{∥s∥}_{H}^{2}{∥\dot{s}∥}_{H}^{2}}. \end{array}$$

## Appendix B. Fisher Information Matrix for ToA and AoA (Multipath Propagation)

In this section, we derive the FIM elements for the case of an arbitrary number of specular MPCs, including the possible path-overlap between specular MPCs. Inserting the likelihood function for the path-overlap case (Equation (45)) into Equation (7), the FIM elements on the main-diagonal are

Jϕkϕκ,m=2Reamk∗amκDm(ϕk)Dm(ϕκ)(2πfc)2smk,smκHm+s˙mk,s˙mκHmJτkτκ,m=2Reamk∗amκ(2πfc)2smk,smκHm+s˙mk,s˙mκHmJRαkRακ,m=JIakIaκ,m=2smk,smκHm.

The off-diagonal FIM elements are

Jϕkτκ,m=2Dm(ϕk)Reamk∗amκ(2πfc)2smk,smκHm+s˙mk,s˙mκHmJϕkRακ,m=2Dm(ϕk)2πfcRejamk∗e−j2πfcτmκsmk,smκHm+Reamk∗e−j2πfcτmκsmk,s˙mκHmJϕkIακ,m=2Dm(ϕk)−2πfcReamk∗e−j2πfcτmκsmk,smκHm+Rejamke−j2πfcτmκsmk,s˙mκHmJτkRακ,m=22πfcRejamk∗e−j2πfcτmκsmk,smκHm+Reamk∗e−j2πfcτmκsmk,s˙mκHmJτkIακ,m=2−2πfcReamk∗e−j2πfcτmκsmk,smκHm+Rejamk∗e−j2πfcτmκsmk,s˙mκHmJRαkIακ,m=0,

where the index *m* can no longer be dropped due to Equation (44). The influence of the array geometry is again captured by Equation (A1).
